# Use of Next-Generation Sequencing and Whole-Exome Sequencing in the Diagnosis of Adult-Onset Familial Intrahepatic Cholestasis: Challenges in Interpreting Variants of Uncertain Significance

**DOI:** 10.3390/diagnostics16152335

**Published:** 2026-07-25

**Authors:** Amalia Conti, Filippo Gabrielli, Simona Ferrari, Alessandro Vaisfeld, Claudia De Masi, Francesco Azzaroli, Fabio Piscaglia, Giovanni Vitale

**Affiliations:** 1 Medical Genetics Unit, IRCCS Azienda Ospedaliero-Universitaria di Bologna, 40138 Bologna, Italy; 2 Internal and Metabolic Medicine, Department of Medical and Surgical Sciences for Children & Adults, AOU of Modena, University of Modena and Reggio Emilia, 41126 Modena, Italy; 3 SSD Biologia e Medicina Molecolare, IRCCS Azienda Ospedaliero-Universitaria di Bologna, 40138 Bologna, Italy; 4 Gastroenterology Unit, IRCCS Azienda Ospedaliero-Universitaria di Bologna, 40138 Bologna, Italy; 5 Department of Medical and Surgical Sciences (DIMEC), University of Bologna, 40138 Bologna, Italy; 6 Division of Internal Medicine, Hepatobiliary and Immunoallergic Diseases, IRCCS Azienda Ospedaliero-Universitaria di Bologna, 40138 Bologna, Italy; 7 Internal Medicine Unit for the Treatment of Severe Organ Failure, IRCCS Azienda Ospedaliero-Universitaria di Bologna, 40138 Bologna, Italy

**Keywords:** adult-onset cholestasis, cryptogenic cholestasis, PFIC, genetic sequencing, next-gGeneration sequencing, whole-eExome sequencing, variant of uncertain significance

## Abstract

Progressive Familial Intrahepatic Cholestasis (PFIC) is a rare liver disorder that, although typically present in childhood, can also occur in adulthood. Early diagnosis is crucial for appropriate clinical management, prognostic assessment, and therapeutic decision-making; however, it remains challenging due to phenotypic variability and the frequent identification of genetic variants of uncertain significance (VUS). Advances in next-generation sequencing (NGS) and whole-exome sequencing (WES) have improved the detection of disease-associated variants, revealing that the same genetic variants—often in a heterozygous state—may lead to adult-onset cholestatic disease, with milder or atypical presentations, while still conferring a significant risk of progressive liver injury and related complications. To assess the diagnostic yield and clinical utility of NGS panels and WES in adults with suspected PFIC or unexplained cholestatic liver disease, focusing on variant interpretation, emerging disease-associated genes, and clinical significance of VUS. A literature review was conducted to assess molecular diagnostics in adult cryptogenic cholestasis, with a focus on studies employing targeted NGS panels and WES. Selected studies were examined for diagnostic yield, variant classification, interpretive challenges, including VUS, and integration with clinical data. Data on patient demographics, sequencing techniques, and variant interpretation strategies were extracted to evaluate the current capabilities and limitations of genomic testing. From an initial search of 211 publications, 15 studies met the inclusion criteria, comprising five large adult cohorts and ten single-patient or trio-based reports. Across 72 patients, sequencing technologies identified 75 pathogenic or likely pathogenic variants, predominantly missense mutations, demonstrating high genetic heterogeneity. The most implicated genes included *ABCB4*, *ABCB11*, *ATP8B1*, *TJP2*, and *USP53*, with diagnostic yields ranging from 13.2% in large heterogeneous cohorts to substantially higher rates in carefully selected individual cases. VUS were identified in up to 67% of cases, highlighting the need for multidisciplinary interpretation integrating clinical, biochemical, and genetic data. Emerging evidence also implicated ciliopathy-associated genes such as *DCDC2*, *NPHP3*, *PKHD1*, *TULP3*, and *TTC21B*, expanding the genetic spectrum of adult cholestasis. Clinically, presentations ranged from mild biochemical abnormalities to progressive cholestasis, with pruritus being a common feature across studies. Comprehensive genetic testing significantly enhances diagnostic accuracy, informs prognosis, and guides individualized management in adults with cryptogenic cholestasis. Emerging evidence suggests that ciliopathy-associated genes may contribute to the genetic architecture of adult cholestatic disorders, further expanding the spectrum of disease-associated genes. However, the high prevalence of VUS remains a major challenge, highlighting the need for functional studies, longitudinal follow-up, and improved variant interpretation frameworks. As genomic technologies and analytical approaches continue to evolve, genetic testing is expected to play an increasingly central role in precision hepatology.

## 1. Introduction

Progressive Familial Intrahepatic Cholestasis (PFIC) constitutes a genetically heterogeneous group of autosomal disorders that typically present in infancy or early childhood, characterized by severe clinical features such as pruritus, jaundice, malabsorption, and rapid progression to fibrosis, cirrhosis, and often end-stage liver disease [1,2].

The PFIC genes display a wide spectrum of variant types: missense, nonsense, splice-site, small insertions/deletions (indels), frameshift, and, in some cases, larger deletions. The severity of the phenotype is often correlated with the type of variant: null alleles (nonsense, frameshift, severe splice variants) tend to cause more severe disease, earlier onset, and more rapid progression. Missense variants may allow residual function and are relatively more frequent in milder or intermittent phenotypes such as low phospholipid-associated cholelithiasis (LPAC), benign recurrent intrahepatic cholestasis (BRIC), or intrahepatic cholestasis of pregnancy (ICP) [1,3].

Although PFICs are classically autosomal recessive, requiring biallelic pathogenic (P) variants, accumulating evidence shows that heterozygous variants in PFIC genes can contribute to disease in adults or adolescents, resulting in milder phenotypes, later onset, and sometimes clinical manifestation only under stress or environmental modifiers. For example, in a cohort of young and adult patients with cryptogenic cholestasis, P or likely pathogenic (LP) variants in *ATP8B1*, *ABCB11*, *ABCB4*, and *TJP2* were found in ~21% of probands; heterozygous variant carriers also exhibited disease, particularly in the presence of clinically relevant features [4,5,6].

The severity, age of onset, biochemical markers, such as bile acids and gamma-glutamyl transferase (GGT) levels, histological findings, and the risk of complications, including the development of fibrosis, cirrhosis, and malignancy, often correlate with the nature of the genetic defects. Null variants produce earlier, more severe disease; missense or partial loss-of-function variants often result in delayed or attenuated phenotypes. Even for the same genetic variants, clinical presentation can vary: some individuals may remain asymptomatic for many years, whereas others may present with chronic cholestasis, gallstones (LPAC), or elevated cholestatic liver enzymes without jaundice or pruritus until adulthood. This variability complicates clinical suspicion and genotype–phenotype correlation. The literature shows that adult patients sometimes harbor only heterozygous variants but manifest cholestatic disease under triggers, such as medications, pregnancy, or other liver insults [4].

The detection of variants of uncertain significance (VUS) is common with next-generation sequencing (NGS) and whole-exome sequencing (WES), especially when testing multiple PFIC genes. Interpreting VUS requires integrating multiple lines of evidence: in silico predictions, evolutionary conservation, structural/functional domain context, allelic frequency in population databases, segregation within families, correlation with biochemical and clinical phenotype, and, when possible, functional assays. Without this, VUS may remain unresolved for a long time, limiting clinical actionability [4,7].

Because many PFIC genes are rare and less well characterized, population data may be limited; many variants have not been previously observed or functionally validated. Recent studies emphasize that combining NGS with detailed phenotypic data, including history of pruritus, bile acid levels, histology, imaging, and non-invasive fibrosis measures, improves confidence in variant interpretation. Moreover, the quantification of liver stiffness, bile acid levels, and other clinical data has been associated with the presence of P and LP variants [1,4].

The introduction of NGS technologies has deeply transformed the diagnostic approach to inherited liver diseases. While Sanger sequencing remains an essential tool for variant validation and targeted analyses, it has been largely replaced as a first-line diagnostic method by high-throughput sequencing approaches. Targeted NGS panels allow the simultaneous analysis of multiple genes associated with cholestatic disorders, providing high sequencing depth and excellent coverage of coding regions and exon–intron boundaries. In contrast, WES allows sequencing of all coding regions of the genome, whereas Whole-Genome Sequencing (WGS) extends the analysis to both coding and non-coding regions [8,9].

**Figure 1:**
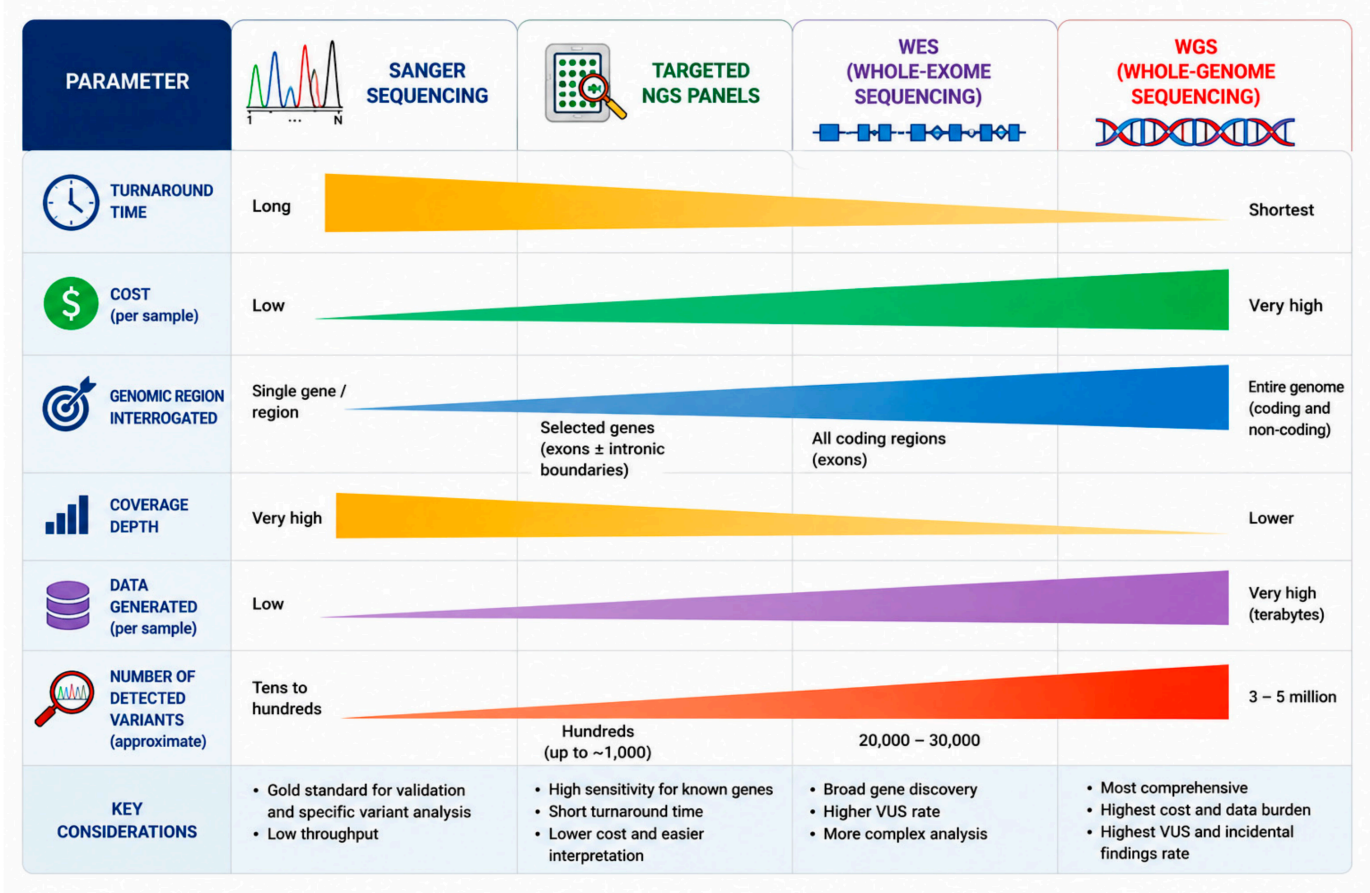
**Advantages and limitations of Sanger sequencing, targeted NGS panels, WES, and WGS in the genetic diagnosis of adult cholestatic disorders.** The figure summarizes the main characteristics of the principal sequencing approaches currently used in clinical and research settings. Targeted NGS panels represent a balanced strategy between diagnostic yield, sequencing depth, cost, and interpretative burden, whereas WES and WGS progressively increase genomic coverage and gene discovery potential at the expense of greater analytical complexity, larger data storage requirements, and higher rates of variants of uncertain significance and incidental findings.

Each approach has specific advantages and limitations. Targeted NGS panels generally provide greater coverage of disease-associated genes, less analytical complexity, shorter interpretation times, and reduced costs. Higher sequencing depth may also improve the detection of low-level mosaic variants. Their targeted design also limits the number of detected variants, thus reducing the burden of VUS and incidental findings. In contrast, WES and WGS offer broader genomic interrogation and the potential to identify novel disease genes; however, they generate substantially larger datasets, require more extensive bioinformatic analyses, and are associated with a greater number of VUS [10] (Figure 1).

In fact, the number of detected variants increases with the length of genomic interrogation. While a targeted cholestasis panel can identify several hundred variants that require filtering and interpretation, WES typically produces tens of thousands of variants and WGS several million variants. Consequently, broader sequencing approaches increase diagnostic opportunities, but it also presents interpretative challenges [11]. However, in adult cohorts with cryptogenic cholestasis, WES plays an important role, especially when targeted panel testing is negative, identifying novel disease-associated genes and novel candidate genes in approximately 25% of singleton analyses. While diagnostic yield may increase by an additional 6 percentage points when trio-based sequencing is performed, involving the proband and both parents. Consequently, trio-WES has become a common strategy for reducing the number of candidate variants and improving variant prioritization and interpretation.

In fact, WES may be reserved for patients with negative panel tests, atypical phenotypes, strong familial clustering, or suspected novel genetic etiologies. WGS is currently limited primarily to research settings or selected unresolved cases due to higher cost, analytical complexity, and data storage requirements [12].

Several studies report a P or LP variant in approximately 20–30% of adult or young adult patients with cryptogenic cholestasis when tested for some PFIC genes (*ATP8B1*, *ABCB11*, *ABCB4*, *TJP2*). Including heterozygous variants increases sensitivity but also complexity. Findings correlate with clinical severity, including fibrosis, bile acid levels, and histologic cholestatic features [4,13,14].

Among patients carrying P/LP variants in the original cohort study, 30% harbored biallelic or multiple P/LP variants in the same gene, whereas 70% carried a single monoallelic P/LP variant, highlighting the important contribution of single heterozygous variants to adult or young-adult cholestatic phenotypes [4]. Early genetic diagnosis can inform prognosis, guide the choice of appropriate therapies—such as pharmacological treatments, biliary diversion, or liver transplantation—identify risk factors, including pregnancy and hepatobiliary malignancy, and influence management decisions, such as monitoring strategies and avoidance of cholestatic insults. This is particularly relevant in the era of ileal bile acid transporter (IBAT) inhibitors. These drugs inhibit bile acid reabsorption in the ileum, leading to increased bile acid excretion in feces and a reduction in the amount of bile acid returning to the liver. This results in reduced itching, slower disease progression, and a lower likelihood of requiring surgery [1].

The therapeutic implications of genetic testing are increasingly evident in patients with variants affecting bile acid transport pathways. Patients carrying *ATP8B1* or *ABCB11* variants may benefit in particular from therapies that reduce the enterohepatic circulation of bile acids, such as IBAT inhibitors. In patients with BSEP deficiency, reducing bile acid levels can alleviate cholestatic injury and pruritus, while *ATP8B1*-related disease may also benefit from limiting bile acid-mediated hepatocellular damage. With the emergence of genotype-specific therapeutic strategies, accurate molecular diagnosis is becoming an essential component of personalized patient management [15]. This highlights the growing role of precision medicine in the management of inherited cholestatic disorders.

Otherwise, disorders associated with defects of tight junction integrity may require different therapeutic approaches. Patients carrying *ABCB4* variants often benefit from ursodeoxycholic acid (UDCA), which remains the mainstay of treatment for MDR3 deficiency and related cholangiopathies. Variants in the *TJP2* and *USP53* genes, involved in the maintenance of hepatocellular tight junctions, may result in increased paracellular bile acid leakage and progressive cholestatic liver injury. Although evidence for genotype-specific therapies in these disorders remains limited, early identification of affected individuals can help with closer monitoring, timely implementation of supportive treatments, and enrollment in future precision-medicine trials. Consequently, the integration of genetic findings into clinical decision-making is expected to play an increasingly important role in optimizing therapeutic strategies for adult patients with inherited cholestatic disorders [15] (Table 1).

Reclassification of variants over time is necessary; periodic review of variants classified as VUS should incorporate new functional, population, or familial data. Genetic counseling must address uncertainty, potential modifiers, and penetrance. Public genetic databases and the guidelines of the American College of Medical Genetics and Genomics (ACMG) are fundamental to this process.

The purpose of this review is to examine:

1. Diagnostic yield and clinical utility of cholestasis-related gene panels, WES and, when available, WGS in adult patients with suspected PFIC or unexplained cholestatic liver disease, including the detection of P/LP variants and VUS.
2. Characterize the genetic landscape of adult-onset cholestatic disorders by analyzing the type, zygosity, and distribution of identified variants, the genes involved, genotype–phenotype correlations, and the emerging contribution of candidate genes, particularly those associated with ciliopathies.
3. Examine the challenges associated with the interpretation of VUS, including the integration of clinical, biochemical, histological, bioinformatic, and functional evidence, and discuss emerging approaches for variant reclassification and their implications for diagnosis, prognosis, and personalized patient management.

## 2. Materials and Methods

A comprehensive literature review was conducted to evaluate current knowledge of molecular diagnostics in adult patients with cryptogenic cholestasis, focusing on studies using targeted NGS panels and WES to investigate the genetic basis of cholestatic liver diseases.

Relevant publications were identified through a manual search of the PubMed, Embase and Scopus databases. The searches included peer-reviewed articles published up to 1 September 2025 and used the following key terms: ‘diagnosis of PFIC in adults’; ‘next-generation sequencing in PFIC patients’; ‘next-generation sequencing in cryptogenic cholestasis’; ‘whole-exome sequencing in PFIC patients’; ‘whole-exome sequencing in cryptogenic cholestasis’; ‘whole-genome sequencing in PFIC patients’ and ‘whole-genome sequencing in cryptogenic cholestasis’. Eligible studies were original research articles reporting molecular findings in adult or young adult patients with cryptogenic cholestasis who underwent genetic testing using targeted NGS panels or WES. Studies involving exclusively pediatric populations or cholestatic conditions of known etiology were excluded.

• The selected studies were subjected to a statistical review with particular attention to the following aspects: diagnostic yield, defined as the proportion of patients carrying the P or LP genetic variant;
• Involved genes, including the spectrum of genes harboring P/LP variants;
• Variant characteristics, including variant types and zygosity of identified pathogenic and likely pathogenic variants;
• Interpretative challenges, including the clinical significance of VUS, incidental findings, and the integration of molecular results with clinical, biochemical and histological data.

Data extraction included collecting detailed demographic and clinical information, sequencing techniques, gene panels used, variant detection rates, and strategies employed for their interpretation. Attention was paid to functional studies, familial segregation analyses, and bioinformatic tools used to support variant classification and assess their clinical relevance. This methodological approach allowed a comprehensive evaluation of the current diagnostic capabilities and limitations of genomic testing in adult cryptogenic cholestasis, providing useful insights to guide future research and clinical practice.

## 3. Results

**Figure 2:**
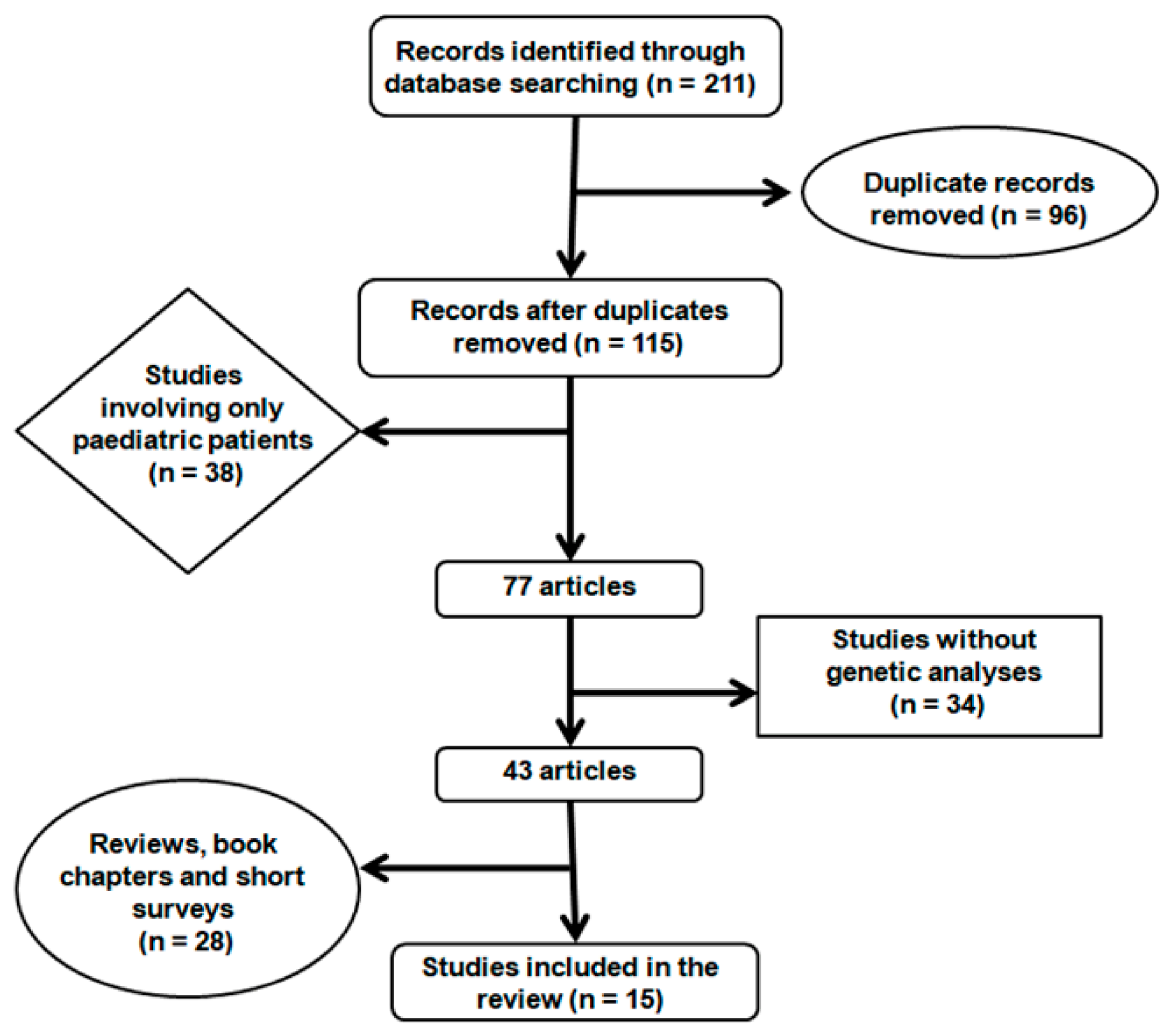
**PRISMA flowchart for study selection.** A total of 211 records were identified through database searching. After removing duplicate records (*n* = 96), 115 publications remained for review. Of these, 100 records were excluded, including studies involving only pediatric patients (n = 38), studies without genetic analyses (n = 34), and publications such as reviews, book chapters, and short surveys (*n* = 28). This refinement significantly reduced the number of eligible articles to 15, ensuring that only those directly relevant to the genetic investigation of cryptogenic cholestasis in adult cohorts using NGS, WES, or WGS technologies were included in this final review (Table 2).

A comprehensive PubMed, Embase and Scopus search yielded a total of 211 scientific publications. From this group of 211 publications, the following were eliminated: articles that appeared more than once in the various searches (96 articles); all articles that included exclusively pediatric patients (38 articles); all publications without genetic analysis (34 articles); and all other publications, such as reviews, book chapters, and short surveys (28 articles) (Figure 2).

Table 2 summarizes the 15 studies included in this review that investigated the genetic basis of cryptogenic cholestasis in adult cohorts using targeted NGS panels, WES, or WGS.

Among the 15 articles included in this review, there were five studies investigating genetic causes in large cohorts of adult patients, while the remaining ten were individual case reports (Table 3).

A total of 541 patients were analyzed, most of whom were under 40. The study population consisted of 55.8% (302/541) women and 44.2% (239/541) men.

**Figure 3:**
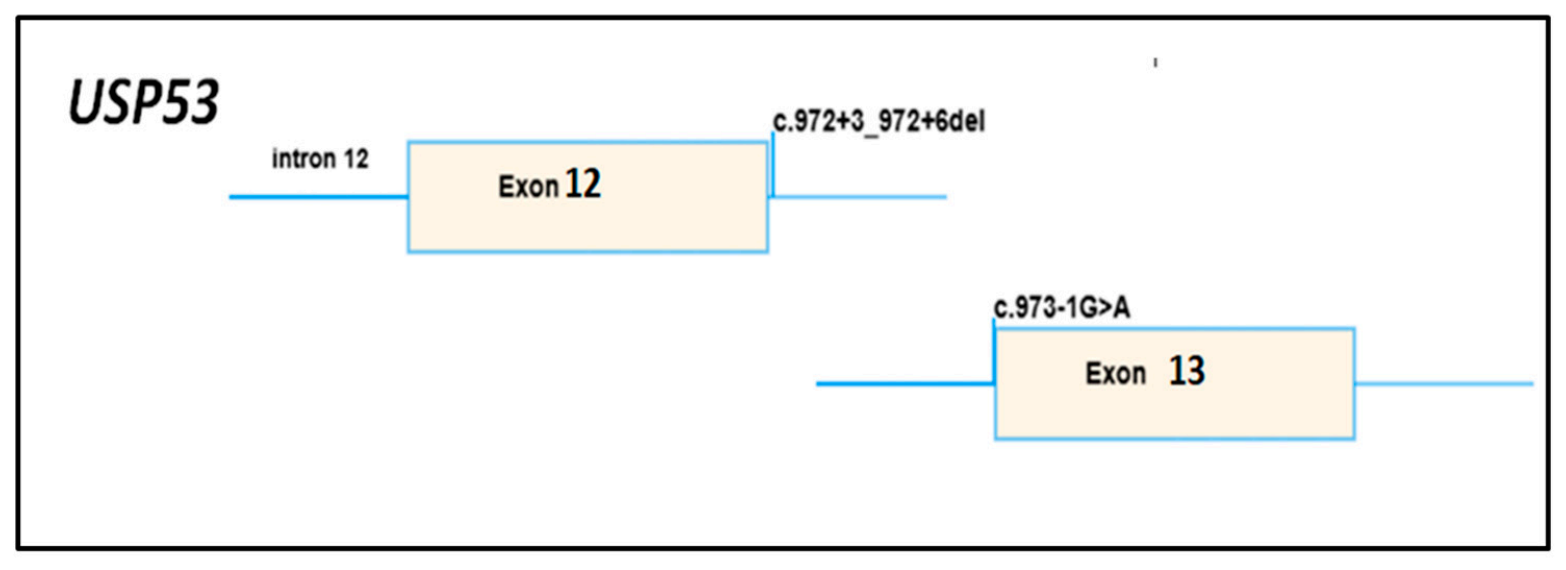
**USP53 variants were identified by WGS**. Schematic representation of the *USP53* variants discovered in the study by Ahn et al. [21]. The canonical splice-site variant c.973-1G>A and the intronic deletion c.972+3_972+6del are both located near exon–intron boundaries and would be expected to be detectable by standard targeted NGS approaches.

The collected studies highlight the growing utility of high-throughput sequencing technologies, including both WES and targeted NGS panels, in elucidating the genetic basis of cholestatic liver diseases across different age groups. The first studies published between 2016 and 2020 used NGS panels, initially small in size (3 or 4 genes) and corresponding to the main PFIC-associated genes known at that time, and subsequently, thanks to the expansion of genetic knowledge, more extensive panels were developed, comprising up to 61 and 76 genes (2020–2022). In 2023, attention gradually shifted towards WES, which has become the most widely used approach for wild-type cases with a strong clinical suspicion of inherited cholestasis. In 2023, Alan S. et al. [21] used WGS as a first-line genetic test in a 21-year-old patient with pruritus that began after taking amoxicillin, who had increased cholestasis markers with GGT levels within normal limits and biopsy findings consistent with cholestasis. However, the two identified variants were in the *USP53* gene, already associated in OMIM with PFIC7. The variants consisted of the canonical splice-site variant c.973-1G>A and an intronic four-nucleotide deletion (c.972+3_972+6del) affecting the donor splice region of exon 12 (Figure 3).

Across large cohort studies, Zheng M. et al. [20] and Scaravaglio M. et al. [17] reported detection rates of 23.8% and 23%, respectively. In contrast, Moral K. et al. [16] identified P or LP variants in only 13.21% of the 53 patients analyzed, while variants of uncertain significance (VUS) were detected in a small subset of patients (approximately 5.7%). Conversely, studies focusing on smaller cohorts or single adult patients frequently report a 100% detection rate of P/LP variants, as observed in multiple case reports and trio analyses [18,19,21], underlining the efficacy of NGS in carefully selected patients with well-characterized phenotypes.

Larger WES studies, such as that by Scaravaglio M. et al. [17], illustrate the complexity of variant interpretation, with a substantial proportion of patients harboring VUS (66.7%), highlighting challenges in clinical correlation and the need for functional validation or familial segregation analysis. Across all studies, the recurrent involvement of key cholestasis-associated genes—including *ABCB4*, *ABCB11*, *ATP8B1*, *TJP2*, and, less frequently, *USP53*—emphasizes their central role in disease pathogenesis.

These data collectively suggest that although sequencing approaches significantly improve diagnostic yield, interpretation remains challenging, particularly in larger, heterogeneous cohorts where VUS are common. Moreover, the variation in P/LP detection rates between large-cohort studies and single-patient studies highlights the importance of patient selection, detailed phenotypic characterization, and the integration of genetic data with clinical findings to achieve optimal diagnostic and prognostic insight.

In addition, the study by Scaravaglio M. et al. [17], conducted on twenty-one adult patients, detected a series of VUS in several genes traditionally implicated in ciliopathies, suggesting that genes such as *DCDC2*, *NPHP3*, *PKHD1* and *TTC21B* may also play a causal role in cholestatic liver diseases.

The collected data highlight the marked phenotypic and genotypic heterogeneity of adult-onset cholestatic disorders, studied by NGS, WES, and WGS. Variants in genes such as *ABCB4*, *ABCB11*, *ATP8B1*, *TJP2*, and *USP53* were associated with a spectrum of clinical presentations, ranging from isolated biochemical cholestasis to advanced liver disease requiring liver transplantation. In particular, patients carrying *ATP8B1* or *ABCB11* variants often showed features reminiscent of PFIC1- or PFIC2-cholestasis, although with later onset and milder progression than in classical pediatric cases, highlighting the concept of intermediate phenotypes. *ABCB4* variants are frequently associated with LPAC, hepatic steatosis, or progressive cholestasis, while *TJP2* variants are associated with cryptogenic cholestasis, pruritus, and, in the most severe cases, cirrhosis and hepatocellular carcinoma.

Clinically, pruritus was the most consistent symptom, even in adults with mild biochemical abnormalities, and laboratory evaluations frequently revealed elevated serum bile acids, bilirubin, and liver enzymes, correlated with the presence of P or LP variants. Across all collected articles, a broad range of genetic variants associated with adult-onset cholestatic disorders was identified.

**Figure 4:**
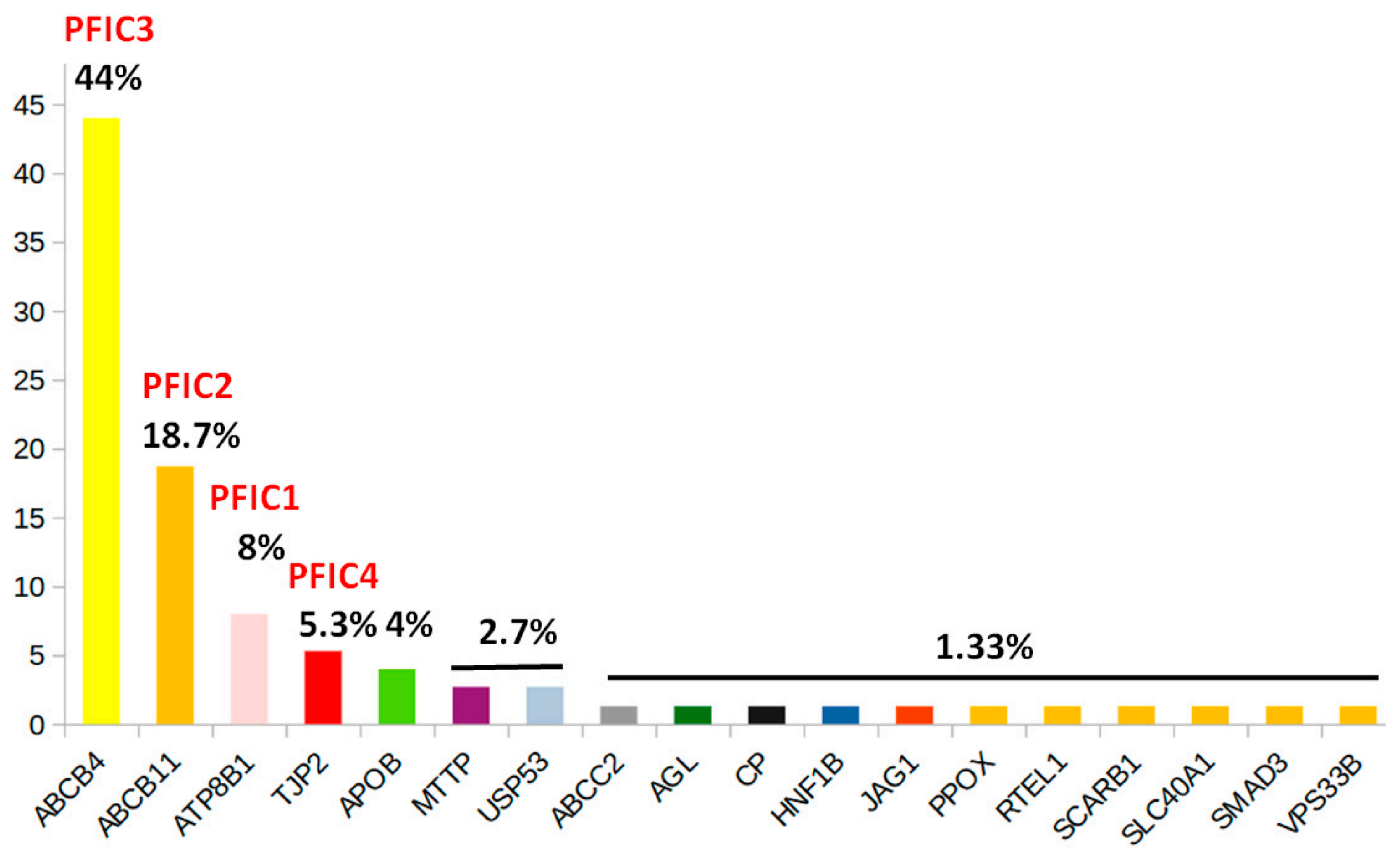
**Distribution of the 75 pathogenic and likely pathogenic variants identified in 72 of the 541 patients included in the reviewed studies.** Variants were found in *ABCB4* (33; 44%), *ABCB11* (14; 18.7%), *ATP8B1* (6; 8%), *TJP2* (4; 5.3%), *APOB* (3; 4%), and *MTTP* (2; 2.7%), with single variants identified in *ABCC2*, *AGL*, *CP*, *HNF1B*, *JAG1*, *PPOX*, *RTEL1*, *SLC40A1*, *SCARB1*, and *SMAD3* (each 1.33%).

The distribution of P and LP variants showed that, out of a total of 541 patients analyzed, 75 variants were identified in 72 subjects. Eighty percent of the variants were in PFIC-associated genes, mainly *ABCB4* (33; 44%), followed by *ABCB11* (14; 18,7%), *ATP8B1* (6; 8%), and *TJP2* (4; 5.3%). The remaining variants were distributed in less frequently involved genes, including *APOB* (3; 4%), *MTTP* (2; 2.7%), *ABCC2*, *AGL*, *CP*, *HNF1B*, *JAG1*, *PPOX*, *RTEL1*, *SLC40A1*, *SCARB1*, and *SMAD3.* Each of the latter genes was represented by a single variant (1.33%) (Figure 4).

**Figure 5:**
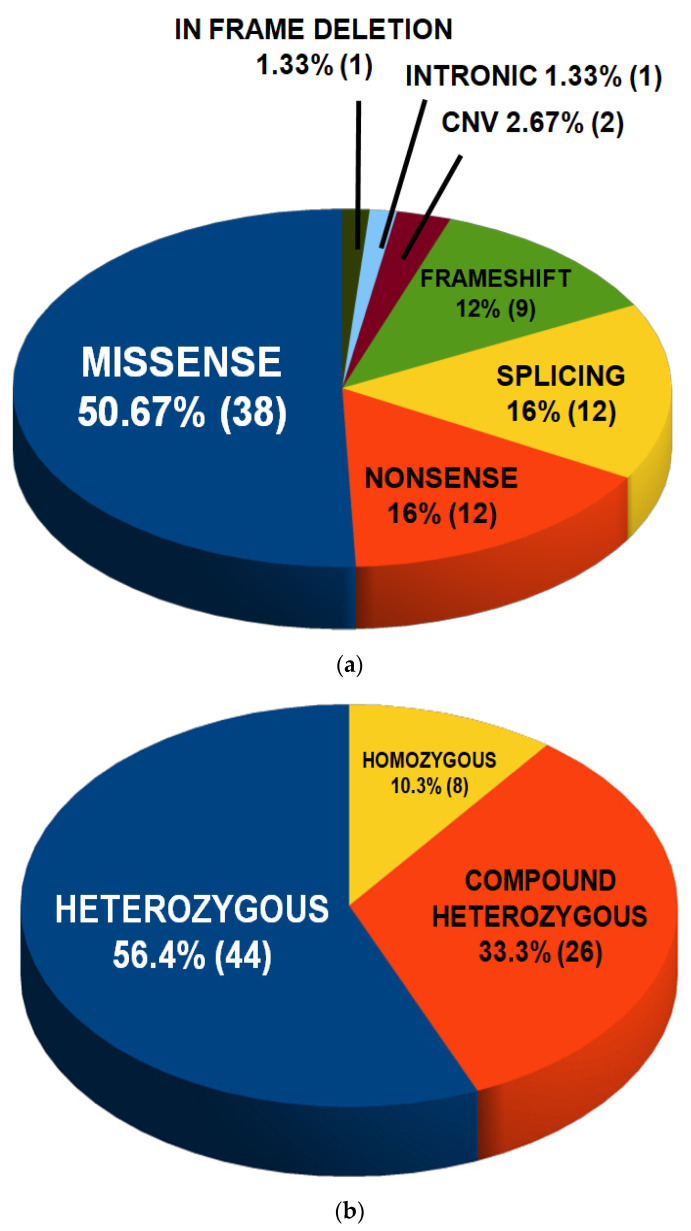
(**a**). **Classification of the 75 pathogenic and likely pathogenic variants identified in the included studies, according to variant type.** Missense variants were the most frequent (38), followed by nonsense (12), splicing (12), and frameshift variants (9). The less represented variant types included 2 copy-number variants (CNVs), 1 in-frame deletion, and 1 intronic variant. (**b**). **Zygosity distribution of pathogenic and likely pathogenic variants in the study cohort.** Distribution of variant zygosity among the 72 patients, showing 44 heterozygous, 26 compound heterozygous, and 8 homozygous variants.

Among the 75 pathogenic or probably pathogenic variants identified, missense variants represented the most frequent category (38/75; 50.7%). There were also 12 nonsense variants (16%), 12 splice variants (16%), and 9 frameshift variants (12%). The remaining variants included two CNVs (2.67%), one in-frame deletion (1.33%), and one intronic variant (1.33%) (Figure 5a).

A total of 75 unique variants were identified. However, when variants were classified by zygosity, the total number increased to 78 because three variants were observed in different zygosity states in unrelated patients. Specifically, *ABCB4* c.2177C>T and *ABCB4* c.1756G>T were identified as both heterozygous variants and components of compound heterozygous genotypes. A similar pattern was observed for *ABCB11* c.3438delA. Among the 72 patients carrying pathogenic or likely pathogenic variants, 44 (56.4%) had a single heterozygous variant, 26 (33.3%) were compound heterozygotes, and 8 (10.3%) were homozygous (Figure 5b).

The complete list of variants, including zygosity, variant type, ACMG classification, and phenotype for each patient analyzed in the individual studies, is shown in Supplementary Table S1.

This distribution highlights the marked genetic heterogeneity underlying adult-onset cholestatic diseases. Overall, these findings highlight the crucial role of comprehensive genetic testing in adult patients with cryptogenic cholestasis, as it provides accurate diagnosis and prognostic information, guides individualized management strategies, and reveals the complex interplay between genotype, phenotype, and disease severity in adult-onset cholestatic liver disease. VUS variants were identified in several genes, highlighting the genetic complexity of adult-onset cholestatic disorders. Among these were *TMEM199* and *FOCAD*, genes previously associated with liver disease and hepatobiliary dysfunction. In particular, the study by Scaravaglio et al. [17] reported VUS in multiple ciliopathy-associated genes, including *CC2D2A*, *DCDC2*, *NPHP3*, *PKD1L1*, *PKHD1*, and *TTC21B*.

Gene symbols associated with VUS in adult-onset cholestatic liver diseases, together with their full names and functional relevance as detected by NGS and WES, are reported in Table 4.

Overall, these findings highlight the marked genetic heterogeneity of adult-onset cholestatic disorders, demonstrating the growing diagnostic value of NGS- and WES-based approaches. At the same time, the high prevalence of variants of uncertain significance underscores the need for careful genotype–phenotype correlation and further functional studies to improve variant interpretation and clinical decision-making.

## 4. Discussion, Clinical Implications and Perspectives

### 4.1. Clinical Utility and Cost-Effectiveness of NGS, WES, and WGS

In recent years, genetic analysis has played an increasingly important role in the diagnosis of patients with familial cholestasis. On the one hand, biopsy is an invasive procedure, and it is not always accepted by all patients; moreover, histological analysis is not always conclusive. On the other hand, the rapid development of various genetic analysis techniques has completely revolutionized clinical testing for rare genetic diseases. A genetic diagnosis is important for choosing the appropriate therapy for the patient, slowing disease progression, and cascade genetic screening of at-risk relatives.

This systematic review summarizes and critically analyzes the current evidence on the use of NGS, WES and WGS to elucidate the genetic basis of adult-onset cryptogenic cholestasis. From an initial pool of 211 articles, only 15 met the inclusion criteria, reflecting the limited but growing number of studies addressing the genetic landscape of cholestatic disorders in adults. These included five large adult cohorts and ten single-patient or trio-based reports, highlighting both the rarity of such studies and the increasing interest in precision hepatology. Therefore, while pediatric pathology is well known and there are many publications on large patient cohorts, there are very few publications on adult-onset patients affected by this disease, mostly case reports, and knowledge on the subject remains limited.

The first thing observed by these studies is that a large-spectrum analysis, such as a WGS, is not always the best choice for a first-level analysis, especially because this type of analysis can generate a huge quantity of data, still difficult to interpret with current knowledge, and therefore not yet ready for routine analysis. So, if the phenotype is suggestive, a simpler analysis with a targeted NGS panel may be more appropriate and sufficient.

In fact, the study by Ahn S. et al. [21] identified two USP53 variants by WGS, a gene already associated with PFIC7. Both variants had neither deep intronic nor complex structural alterations, and both would have been identifiable also using a dedicated NGS panel for hereditary cholestasis. Furthermore, the patient’s clinical phenotype did not appear particularly complex or atypical. These features could have been related to a phenotypic spectrum of low-GGT PFIC and could have been initially addressed by analyzing known genes associated with these forms. In this context, a targeted NGS panel for hereditary cholestasis would likely identify causal variants and answer the diagnostic question. The patient’s clinical phenotype was not complex or atypical; he was a 21-year-old who began experiencing itching after taking amoxicillin, with elevated cholestasis indices except for a normal GGT, and cholestasis on biopsy. This finding suggests that, although WGS provides the most comprehensive genomic analysis, its diagnostic advantage over targeted NGS panels or WES may be limited when variants occur in well-established cholestasis genes.

### 4.2. Diagnostic Yield and Factors Influencing Molecular Diagnosis

The other observation to be made concerns the detection rate, because the two published articles that used WES on large cohorts, Scaravaglio M. et al. [17] and Zheng M. et al. [20], had a detection rate, respectively, of 23.8% and 23%, while Moral K. et al. [16] had a much lower detection rate (13.2%). If initially it could be hypothesized that this could depend on the clinical heterogeneity of the patients analyzed, rather than on the size of the cohort, then it was hypothesized that this could probably depend on how the patients analyzed had been selected, since the two studies with the same number of samples had the same phenotypic groups but very different detection rates (cholestasis, hepatic steatosis, cryptogenic cirrhosis and increased liver enzymes). Scaravaglio M. et al. [17] included 21 patients: 2/21 (9.5%) with recurrent gallstones, later found to harbor *ABCB4* alterations consistent with an LPAC phenotype, 7/21 (33.3%) with intrahepatic cholestasis, and 12/21 (57.1%) with atypical Primary Sclerosing Cholangitis (PSC).

Two patients with PSC with atypical features received a genetic diagnosis. One patient had a heterozygous *PPOX* variant, which led to the diagnosis of variegate porphyria, a rare autosomal dominant disorder caused by a deficiency in protoporphyrinogen oxidase, resulting in the accumulation of porphyrins and their precursors with multisystem manifestations. Although it has been associated with liver enzyme alterations and a risk of cholelithiasis due to high levels of protoporphyrin in bile, the association with sclerosing cholangitis has not yet been described and should therefore be interpreted with caution [17]. In a patient with PSC and hepatic steatosis, WES revealed the presence of a variant in *APOB*, leading to the diagnosis of familial hypobetalipoproteinemia, a known cause of fatty liver disease with a high probability of progression to advanced hepatic fibrosis and primary liver cancer [17].

In these studies, NGS-based panel testing and whole-exome sequencing revealed P and LP variants in key cholestasis-associated genes, most frequently *ABCB4*, *ABCB11*, *ATP8B1*, *TJP2*, and *USP53*, with additional contributions from ciliopathy-related genes such as *DCDC2*, *NPHP3*, *PKHD1*, and *TTC21B* [16,17,18,19,20,21,22,23,24,25,26,27,28]. These findings indicate marked genetic heterogeneity in adult-onset cholestatic liver disease and suggest that diagnostic yield is influenced by patient selection, phenotypic definition, and analytical depth.

### 4.3. Genetic Heterogeneity and Emerging Candidate Genes

Recent studies have increasingly implicated ciliopathy-associated genes, including *DCDC2*, *KIF12*, *TTC21B*, and *TULP3*, in cholestatic liver disease. For example, biallelic truncating variants in *DCDC2*, a gene expressed in cholangiocyte primary cilia and previously known to cause nephronophthisis type 19 [29], have been shown to cause neonatal sclerosing cholangitis and progressive cholestasis, supporting a hepatorenal ciliopathy mechanism [30,31,32,33]. Similarly, homozygous variants in *KIF12* have been identified in patients with high GGT cholestasis, implicating defects in hepatocyte polarity and ciliary function. Moreover, *KIF12* variants have recently been associated with PFIC8, further expanding the genetic spectrum of cholestatic liver diseases [34,35,36]. More recently, compound heterozygous variants in the *TULP3* gene have been identified in patients with neonatal cholestasis and liver fibrosis, highlighting the role of altered ciliary trafficking in disease pathogenesis [37]. These observations expand the genetic spectrum of early-onset and adult-onset cholestatic disorders beyond classical canalicular transport defects and underscore the importance of including ciliopathy genes in diagnostic gene panels for cholestasis, supporting a model in which ciliary dysfunction alters bile duct architecture and hepatocellular polarity.

Although *DCDC2*, *KIF12*, *TTC21B*, and *TULP3* are increasingly recognized as potential contributors to cholestatic liver disease, evidence supporting their role remains limited compared with well-established cholestasis genes such as *ABCB4*, *ABCB11*, *ATP8B1,* and *TJP2*. Current data are largely derived from small cohorts, single case reports, or studies identifying variants through large-scale sequencing approaches, and many of these genes should therefore still be considered candidate genes. Consequently, variants identified in these genes, particularly VUS, require cautious interpretation and, where possible, further functional validation and replication in independent cohorts before being considered a definitive cause of disease. Nonetheless, inclusion of ciliopathy-associated genes in larger cholestasis panels could improve diagnostic yield, particularly in patients presenting with hepatorenal features, ductal plaque malformations, cholestasis with elevated GGT levels, or other clinical manifestations suggestive of ciliopathy. As sequencing technologies become more accessible and more evidence accumulates, the contribution of these genes to the genetic architecture of adult cholestatic disorders is likely to be more clearly defined.

In larger WES cohorts, such as that of Moral K. et al. [16], the detection rate of P/LP and VUS variants was modest. In contrast, smaller series and case reports have achieved higher diagnostic rates, often identifying biallelic variants in canonical cholestasis genes, such as *ABCB4*, *ATP8B1*, *ABCB11* and *TJP2*. These findings suggest that while broad genetic screening improves diagnostic yield, the challenge of variant interpretation, especially VUS, remains significant, particularly in genetically heterogeneous adult populations.

Despite adherence to ACMG guidelines, classification uncertainty has limited direct clinical application. In contrast, studies such as those by Wang Z. et al. [18], Jiao J. et al. [19], Ahn S. et al. [21], and Qiao F. et al. [22] have not reported clinically relevant VUS, instead identifying well-characterized P variants that have provided direct insight into diagnosis and management, including the precise classification of PFIC subtypes (PFIC1, PFIC3, and PFIC7).

### 4.4. Molecular Mechanisms Underlying Adult-Onset Cholestatic Phenotypes

The increasing use of NGS and WES has shown that adult-onset cholestatic disorders are frequently associated with variants that partially preserve protein function, resulting in milder and later-onset phenotypes compared with classical pediatric PFIC. *ATP8B1* encodes FIC1, a protein involved in maintaining canalicular membrane phospholipid asymmetry. Hypomorphic variants can partially preserve activity, leading to impaired membrane stability and mild or intermittent cholestatic phenotypes that manifest later in life [38].

*ABCB11* encodes the bile salt export pump (BSEP), which contains ATP-binding and transmembrane domains, both of which are essential for bile acid transport. Variants affecting ATP-binding domains can impair transporter activity, while variants located in transmembrane domains can alter protein folding, stability, or trafficking to the canalicular membrane. Residual BSEP activity may explain the later onset and variable clinical presentation observed in adult patients [39].

*ABCB4* encodes MDR3, a phosphatidylcholine transporter that protects the biliary epithelium from bile acid toxicity. Missense variants can reduce canalicular expression or phospholipid secretion without completely abolishing protein function, leading to chronic biliary injury and adult-onset cholestatic disease [40].

**Figure 6:**
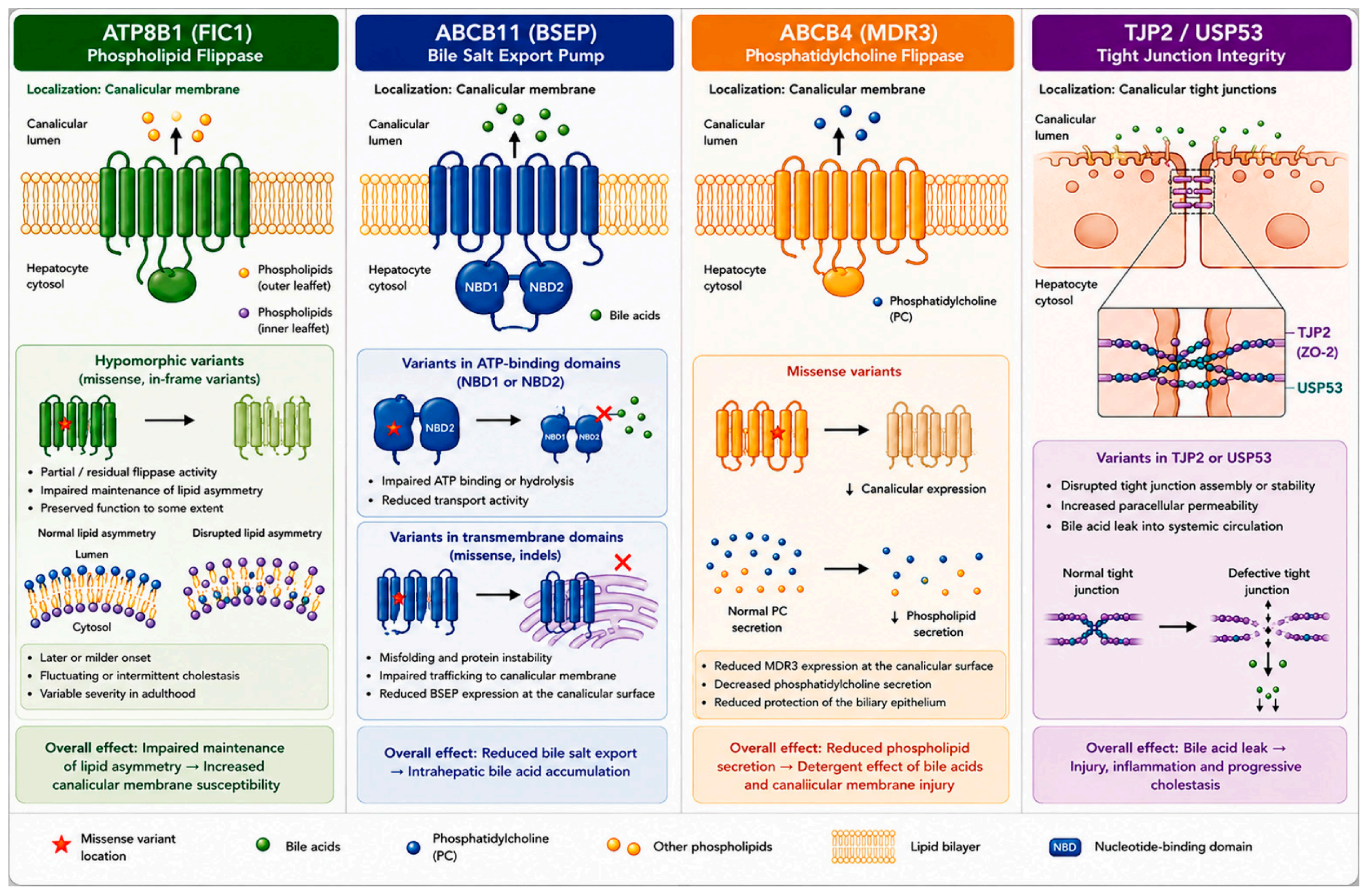
Molecular mechanisms underlying adult-onset cholestatic phenotypes. Schematic representation of the main proteins involved in inherited cholestatic disorders and the mechanisms by which partial functional impairment may contribute to adult-onset disease. *ATP8B1* (FIC1) maintains canalicular membrane phospholipid asymmetry, while *ABCB11* (BSEP) mediates bile acid export through ATP-binding and transmembrane domains. *ABCB4* (MDR3) promotes phosphatidylcholine secretion into bile, protecting the biliary epithelium from bile acid toxicity. *TJP2* and *USP53* are involved in maintaining the integrity of hepatocellular tight junctions. Variants affecting these proteins can impair bile acid transport, canalicular membrane stability, phospholipid secretion, or tight-junction function, resulting in chronic cholestatic liver injury. Residual protein activity associated with hypomorphic or heterozygous variants may contribute to the milder and delayed clinical phenotypes frequently observed in adult patients.

*TJP2* and *USP53* are involved in maintaining hepatocellular tight junctions. Variants affecting these proteins can increase paracellular leakage of bile acids into the liver parenchyma, promoting chronic inflammation and fibrosis. Partial preservation of protein function may contribute to the relatively mild and heterogeneous phenotypes frequently observed in adults [41] (Figure 6).

These observations suggest that adult-onset cholestatic disorders often result from partial defects in bile acid transport, canalicular membrane integrity, or tight junction function, rather than from complete loss of protein activity.

### 4.5. Adult Versus Pediatric Cholestatic Phenotypes

Phenotypically, patients carrying *ABCB11* or *ATP8B1* variants often present PFIC2- or PFIC1-like features, with later onset and milder progression than their pediatric counterparts [16,22,28], suggesting an intermediate or atypical adult phenotype. *ABCB4* variants have been associated with LPAC, hepatic steatosis, or progressive cholestasis [4,24,26], while *TJP2* variants have been associated with cryptogenic cholestasis or cirrhosis, occasionally complicated by hepatocellular carcinoma [27,42]. *USP53* variants have been linked to low-GGT cholestasis (PFIC7) with mild presentation in adults [21,43]. Across all studies, pruritus was the most consistent clinical symptom, even in the presence of mild biochemical disease, emphasizing the need for genetic testing in adults with unexplained cholestasis.

An important difference between pediatric and adult cholestatic disorders emerges from both zygosity and the type of variants identified. Pediatric PFIC typically presents during the neonatal period or early childhood and is most associated with homozygous or compound heterozygous variants that result in severe loss of protein function. In contrast, adult patients more frequently harbor a single heterozygous variant, often of the missense type. Indeed, most pathogenic and likely pathogenic variants identified in this review were missense variants. Unlike nonsense, frameshift, or canonical splicing variants, which frequently lead to truncated or non-functional proteins, missense variants often preserve partial protein activity despite affecting conserved residues or critical functional domains. Consequently, adult-onset phenotypes may be associated with a lower degree of functional impairment, resulting in residual protein activity, milder clinical manifestations, incomplete penetrance, and delayed disease onset. Overall, these findings suggest that adult cholestatic disorders frequently arise from partial disruption of key hepatobiliary pathways rather than complete loss of protein function.

### 4.6. Challenges in the Interpretation of Variants of Uncertain Significance

It is important to note that the proportion and management of VUS differed markedly among studies (Table 3). While Moral, K et al. [16] and Zheng, M. et al. [20] reported relatively low VUS rates (5.7% and 11.5%, respectively), Scaravaglio M. et al. [17] reported about two-thirds of variants as VUS, other groups such as Wang Z. et al. [18], Jiao J. et al. [19], Qiao F. et al. [22] and Suzuki H. et al. [23], reported none, reflecting differences in analytical rigor, variant databases, and multidisciplinary review practices. Functional validation, segregation studies, and clinical phenotype integration remain crucial for reclassifying uncertain variants and refining genotype–phenotype correlations. The summary of these findings highlights the substantial heterogeneity both in the proportion of VUS detected and in how variant classification is performed and applied clinically in cohorts of patients of adult-onset cholestatic liver disease analyzed by NGS, WES, or WGS. Some studies report a relatively high proportion of VUS, whereas others report none or only a few. Methodological stringency, multidisciplinary team involvement, and interpretative criteria all contribute to this variability. Clinically, when pathogenic P or LP variants are identified, diagnosis, management, family counseling, and prognosis can be significantly improved. In contrast, VUS remain a challenge, highlighting the need for ongoing functional and segregation analyses, close clinical monitoring, and recognition that such variants often cannot yet guide definitive management decisions. These observations align with broader challenges in medical genetics in interpreting and translating uncertain genomic findings.

Variants of uncertain significance or VUS are alterations that are difficult to classify or those of class 3 for the ACMG classification. Currently, geneticists lack guidelines on when to report them and when not to. The reporting of VUS remains controversial, and current practices vary among laboratories and institutions. Some laboratories prefer not to report VUS to avoid potential misinterpretation, incorrect diagnoses and inappropriate therapy for the patient, whereas others report them to facilitate clinical correlation and future re-evaluation [44].

First, genetic data must always be contextualized. Data reported must correlate with the phenotype. Then, they should be segregated within the family, regardless of phenotypic overlap, to determine whether the variant can be classified as likely benign or could actually be implicated in the disease. The phenotype of patients with variants in the same gene should always be compared by consulting PubMed articles or using open-source databases such as Gene Matcher, genotype–phenotype matching, or private databases enriched and developed over the years.

Given the controversial clinical interpretation of VUS in cholestatic genetic disease, such variants should ideally be re-evaluated every 2-5 years. This interval allows for the incorporation of new scientific evidence, expanded population databases, or updated functional studies that may support reclassification. Available data indicate that a proportion of VUS—reported in some series to be up to approximately 15%—may be reclassified as P or LP variants over time. Since international guidelines suggest that VUS should not be used for clinical decision-making until reclassification, it is crucial that clinicians and genetic laboratories maintain structured follow-up and do not lose sight of longitudinal evaluation [45,46].

### 4.7. Emerging Approaches for VUS Interpretation

#### 4.7.1. Advanced Bioinformatic Prediction Tools

The interpretation of VUS is rapidly evolving thanks to advances in computational biology and artificial intelligence. In addition to traditional prediction tools such as SIFT and PolyPhen-2, newer algorithms including REVEL, CADD, SpliceAI, and AlphaMissense provide increasingly accurate prediction of variant pathogenicity. In particular, SpliceAI enables the identification of cryptic splice-site alterations located outside of canonical splice junctions, while AlphaMissense applies deep-learning methodologies to predict the functional consequences of amino acid substitutions. These tools are becoming increasingly important for prioritizing variants that should have been further studied through experimental investigation [47].

#### 4.7.2. Functional Validation Studies

Functional validation remains the gold standard for VUS interpretation and reclassification. Recently, several experimental approaches have been applied in inherited cholestatic disorders, including minigene assays for the evaluation of splicing defects, protein localization studies, bile acid transport assays, and protein expression and stability analyses. Furthermore, hepatocyte-like cells, such as patient-derived induced pluripotent stem cells (iPSCs), have emerged as valuable models for investigating the biological consequences of disease-associated variants and assessing their impact on hepatocellular function [48].

#### 4.7.3. Animal Models

Animal models have become powerful tools for evaluating the pathogenicity of candidate cholestasis genes. Zebrafish and mouse models allow for the assessment of bile duct development, bile acid homeostasis, hepatobiliary architecture, and disease progression in vivo. These systems can provide important evidence to support or refute the pathogenic role of newly identified variants and candidate genes [49].

#### 4.7.4. Multidisciplinary Integration

**Figure 7:**
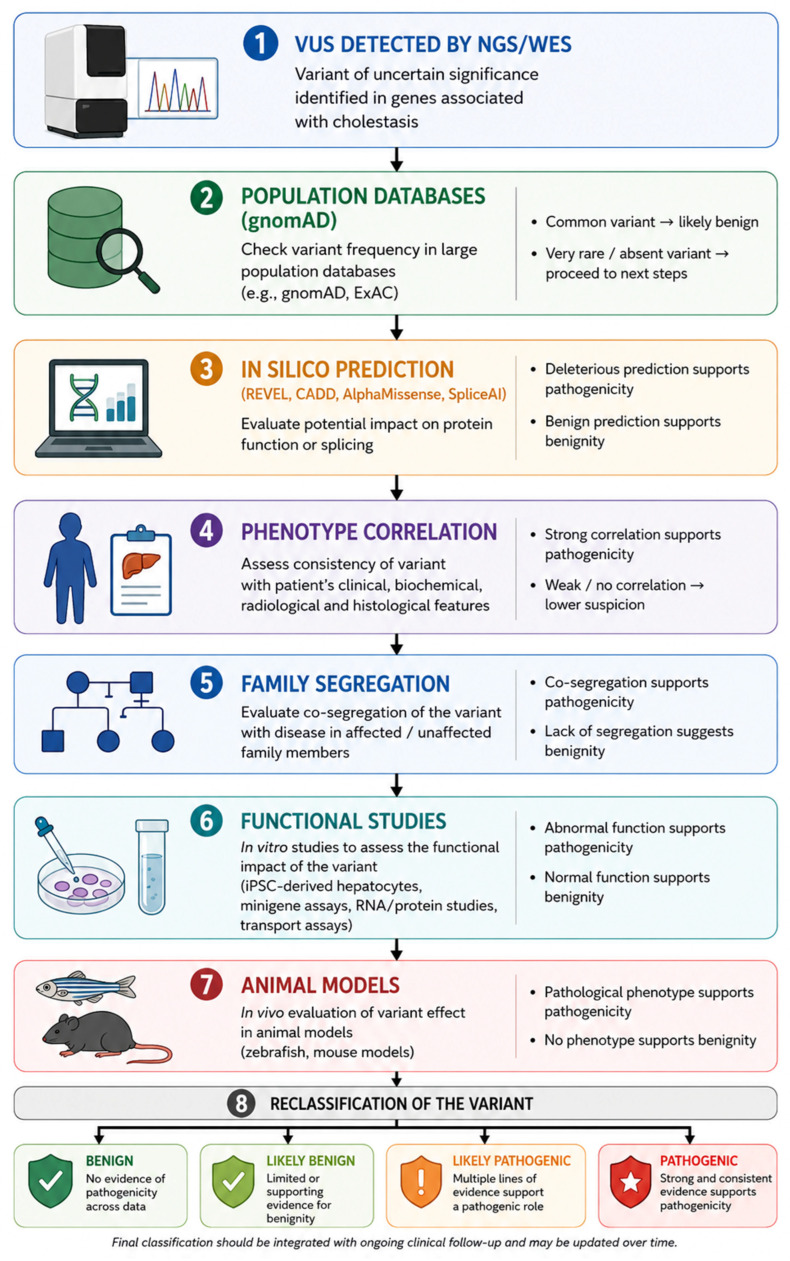
**Framework for the interpretation and reclassification of VUS in adult cholestatic liver disease.** Proposed workflow for the evaluation of variants of uncertain significance identified by NGS or WES. Variant interpretation begins with assessment of allele frequency in population databases, followed by in silico prediction using tools such as REVEL, CADD, AlphaMissense, and SpliceAI. Subsequent steps include correlation with clinical, biochemical, and histological phenotypes, familial segregation analysis, functional validation studies, and evaluation in animal models. The integration of these complementary lines of evidence may support variant reclassification according to established criteria, leading to classification as benign, likely benign, likely pathogenic, or pathogenic. This framework highlights the multidisciplinary approach required for accurate interpretation of VUS in inherited cholestatic disorders.

Future classification of VUS will likely rely on integrated approaches combining genomic data, advanced bioinformatic predictions, transcriptomic analyses, functional studies, familial segregation data, and longitudinal clinical follow-up. These multidisciplinary strategies are expected to improve diagnostic accuracy, facilitate variant reclassification, and support the implementation of precision medicine for adult cholestatic liver diseases [50] (Figure 7).

### 4.8. Proposed Diagnostic Workflow for Adult Cryptogenic Cholestasis

**Figure 8:**
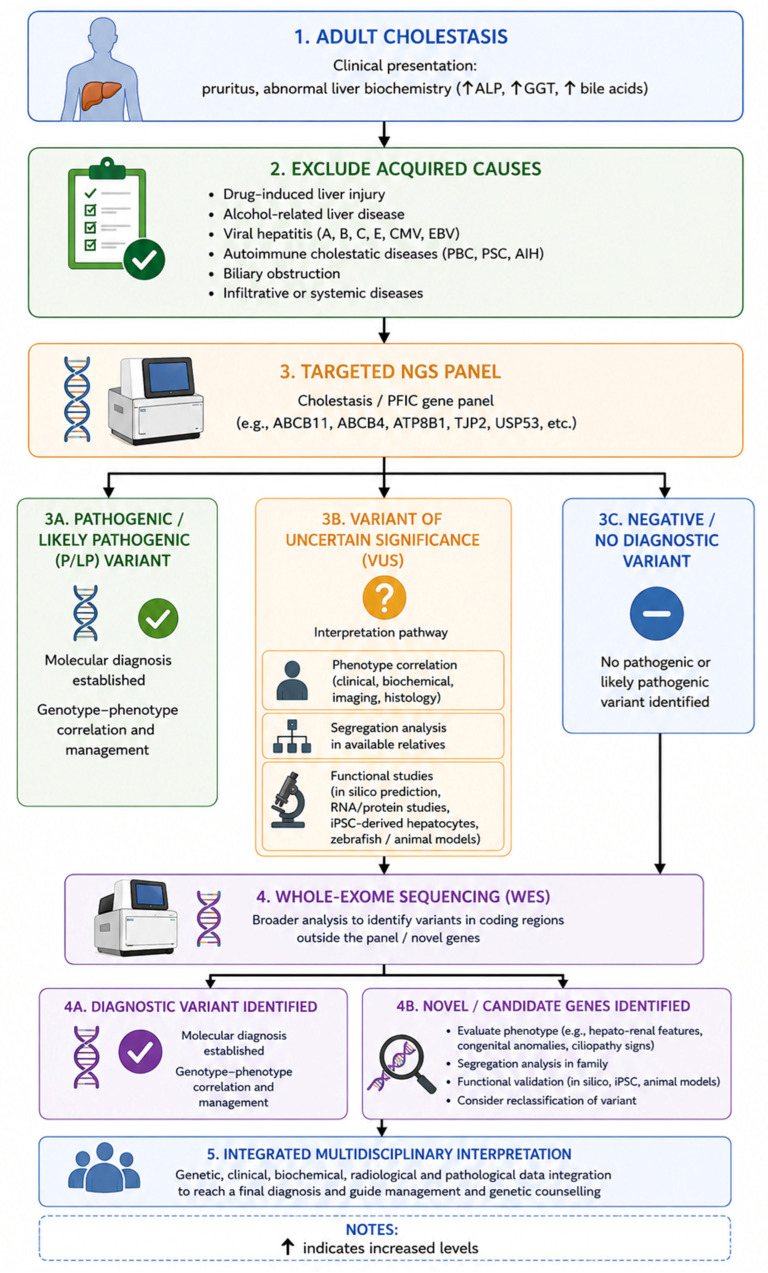
**Proposed diagnostic workflow for adult cryptogenic cholestasis.** Proposed diagnostic algorithm for the evaluation of adult patients presenting with cryptogenic cholestatic liver disease. Following the exclusion of acquired causes of cholestasis, targeted NGS panels represent the preferred first-line molecular approach because of their high coverage of established cholestasis-associated genes and a favorable cost-effectiveness profile. The identification of pathogenic or likely pathogenic variants may establish a molecular diagnosis, while VUS variants require integration with clinical, biochemical, histological, and familial data, together with functional studies when available. In patients with negative panel testing, atypical phenotypes, strong familial clustering, or suspected novel genetic etiologies, WES may identify additional candidate genes.

The diagnostic approach to adult cryptogenic cholestasis has evolved considerably with the increasing availability of genomic technologies. Based on the evidence reviewed, a practical diagnostic workflow can be proposed, integrating clinical evaluation, targeted NGS panels, and WES for selected unresolved cases (Figure 8).

### 4.9. Future Perspectives: Toward a Genomics-Informed Hepatology

The evolution of molecular diagnostics over the last decade has profoundly changed the evaluation of inherited cholestatic liver diseases. Whereas Sanger sequencing was once the only available tool for genetic diagnosis, clinicians now have access to increasingly comprehensive genomic technologies, including targeted NGS panels, WES, and WGS. However, the challenge is no longer simply generating more genomic data but rather selecting the most appropriate testing strategy and accurately interpreting the resulting findings.

Overall, this review underscores the critical role of comprehensive genetic testing in adults with cryptogenic cholestasis. By enabling precise diagnosis, informing prognosis, guiding individualized management, and identifying novel genotype–phenotype relationships, NGS technologies have transformed the diagnostic landscape of adult cholestatic liver disease. Nevertheless, continued refinement of bioinformatics tools, variant classification criteria, and collaborative genotype–phenotype databases will be essential to maximize their clinical utility.

The clinical value of these technologies depends not only on their diagnostic capabilities but also on the integration of molecular results with clinical, biochemical, histological, and familial information. In adult cholestatic liver disease, genetic testing should not replace clinical reasoning; instead, it should refine and strengthen it. As sequencing technologies continue to evolve and variant interpretation becomes increasingly sophisticated, hepatology is progressively moving towards a genomics-informed model of care, in which molecular and clinical data are integrated to improve diagnostic precision, prognostic assessment, and personalized therapeutic decision-making.

## 5. Conclusions

This review highlights the growing role of genomic testing in the diagnosis of adult-onset cryptogenic cholestasis. Targeted NGS panels currently represent the most practical first-line approach because of their favorable balance between diagnostic yield, coverage of established cholestasis genes, cost-effectiveness, and reduced burden of variants of uncertain significance. WES remains particularly valuable in unresolved cases, atypical phenotypes, or when novel genetic etiologies are suspected. The studies reviewed confirm the marked genetic heterogeneity of adult cholestatic liver disease, with pathogenic variants most frequently identified in *ABCB4*, *ABCB11*, *ATP8B1*, *TJP2*, and *USP53*. Emerging evidence also suggests a potential role for ciliopathy-associated genes, including *DCDC2*, *KIF12*, *TULP3*, and *TTC21B*, although additional functional and replication studies are required before several of these genes can be considered fully established causes of disease. Compared with pediatric PFIC, adult-onset cholestatic disorders are more frequently associated with heterozygous and missense variants that preserve partial protein function, resulting in milder phenotypes, incomplete penetrance, and delayed clinical presentation. These observations emphasize the importance of integrating molecular findings with clinical, biochemical, histological, and familial data. Although VUS remain a major challenge, advances in bioinformatic prediction tools, functional genomics, patient-derived cellular models, and animal studies are progressively improving variant interpretation and reclassification. Future diagnostic strategies will increasingly rely on multidisciplinary approaches that combine genomic, functional, and clinical evidence. Overall, the field is evolving from a phenotype-driven approach toward a genomics-informed model of hepatology, in which molecular diagnosis not only improves diagnostic accuracy but also supports prognostic assessment, personalized therapeutic strategies, and precision medicine for adult patients with inherited cholestatic liver disease.

## Figures and Tables

**Table 1:** **Therapeutic implications of the main genes involved in adult-onset cholestatic disorders.** The table summarizes the main pathogenic mechanisms associated with the most frequently implicated cholestasis genes and their current or potential therapeutic implications. While *ATP8B1*- and *ABCB11*-related diseases may benefit particularly from therapies that reduce the enterohepatic circulation of bile acids, such as ileal bile acid transporter (IBAT) inhibitors, *ABCB4*-related disease remains more responsive to ursodeoxycholic acid (UDCA)-based strategies. For *TJP2*- and *USP53*-related disorders, evidence supporting genotype-specific therapies remains limited, underscoring the need for further translational research and precision-medicine approaches.

Gene	Protein	Main Pathogenic Mechanism	Potential Therapeutic Implications
***ATP8B1***	FIC1	Impaired maintenance of canalicular membrane phospholipid asymmetry and bile acid homeostasis	IBAT inhibitors, supportive management, biliary diversion, liver transplantation in advanced disease
***ABCB11***	BSEP	Impaired bile salt export leading to intrahepatic bile acid accumulation	IBAT inhibitors, biliary diversion, liver transplantation in advanced disease
***ABCB4***	MDR3	Reduced phosphatidylcholine secretion into bile with increased bile duct toxicity	UDCA remains the first-line treatment; liver transplantation in advanced disease
***TJP2***	Tight Junction Protein 2	Defective tight-junction integrity with paracellular bile acid leakage	Supportive management, fibrosis surveillance, and potential future targeted therapies
***USP53***	USP53	Tight-junction dysfunction and impaired epithelial barrier function	Supportive management and potential future precision-medicine approaches

**Table 2:** **Summary of 15 studies investigating the genetic basis of cryptogenic cholestasis in adult cohorts using targeted NGS panels, WES or WGS, included in this review.**

Authors and Year	Article Title	Reference
Moral K, Kayhan G, Duzenli T. et al.(2025)	Exome Sequencing in Adults with Unexplained Liver Disease: Diagnostic Yield and Clinical Impact	[16]
Scaravaglio M, Ronzoni L, Cristoferi L et al. (2025)	Diagnostic Yield of Whole-Exome Sequencing in Adult-onset Cholestatic Liver Disease	[17]
Wang Z, Qi H, Wei X et al. (2025)	Clinical and genetic analysis of a Chinese pedigree with autosomal recessive familial intrahepatic cholestasis type I due to a novel variant of ATP8B1 gene	[18]
Jiao J, Morotti R, Shafizadeh N et al. (2025)	Expanding the Spectrum of Progressive Familial Intrahepatic Cholestasis: A Report of 3 Cases	[19]
Zheng M, Hakim A, Konkwo C et al. (2023)	Advancing diagnosis and management of liver disease in adults through exome sequencing	[20]
Ahn S, Choi J, Jeong SH (2023)	The First Korean Adult Case of Progressive Familial Intrahepatic Cholestasis Type 7 with Novel USP53 Splicing Variants by Next-Generation Sequencing	[21]
Qiao F, Ren F, Lu W et al. (2023)	A Female of Progressive Familial Intrahepatic Cholestasis Type 3 Caused by Heterozygous Mutations of ABCB4 Gene and Her Cirrhosis Improved After Treatment of Ursodeoxycholic Acid: A Case Report	[22]
Suzuki H, Arinaga-Hino T, Sano T et al. (2022)	Case Report: A Rare Case of Benign Recurrent Intrahepatic Cholestasis-Type 1 With a Novel Heterozygous Pathogenic Variant of ATP8B1	[23]
Zhu H, Wang S, Li L et al. (2022)	Case Report: A Rare Case of Young Adult Progressive Familial Intrahepatic Cholestasis-Type 3 with a Novel Heterozygous Pathogenic Variant of ABCB4	[24]
Cheng J, Gong L, Mi X et al. (2022)	Case series of progressive familial intrahepatic cholestasis type 3: Characterization of variants in ABCB4 in China	[25]
Nayagam JS, Foskett P, Strautnieks S et al. (2022)	Clinical phenotype of adult-onset liver disease in patients with variants in ABCB4, ABCB11, and ATP8B1	[5]
Goubran M, Aderibigbe A, Jacquemin E et al. (2020)	Case Report: Progressive Familial Intrahepatic Cholestasis Type 3 with Compound Heterozygous ABCB4 Variants Diagnosed 15 Years After Liver Transplantation	[26]
Wei CS, Becher N, Friis JB et al. (2020)	New Tight Junction Protein 2 Variant Causing Progressive Familial Intrahepatic Cholestasis Type 4 in Adults: A Case Report	[27]
Vitale G, Gitto S, Raimondi F et al. (2018)	Cryptogenic Cholestasis in Young and Adults: ATP8B1, ABCB11, ABCB4, and TJP2 Gene Variants Analysis by High-Throughput Sequencing	[4]
Vitale G, Pirillo M, Mantovani V et al. (2016)	Bile Salt Export Pump Deficiency Disease: Two Novel, Late Onset, ABCB11 Mutations Identified by Next-Generation Sequencing	[28]

**Table 3:** **Diagnostic yield of PFIC gene panels, WES and WGS in adult patients.** * Ciliopathy-related genes.

Reference	No. of Patients	Type of Test	Pathogenic (P)/Likely Pathogenic (LP) Variants % (n)	VUS % (n)	Genes with P/LP Variants	Genes with VUS
[16]	53	WES	13.2% (7/53)	~5.7% (3/53)	*ABCB4*, *AGL*, *CP*, *MTTP*, *VPS33B*	*APOB*, *FOCAD*, *KLF11*, *TMEM199*
[17]	21	WES	23.8% (5/21)	66.7% (14/21)	*ABCB4*, *ABCC2*, *PPOX*, *APOB*	*ABCB11*, *ABCB4*, *ABCC2*, *ABCC4*, *APOB*, *ATP8B1*, *DCDC2* *, *HFE*, *MBOAT7*, *NPHP3* *, *PKHD1* *, *PPOX*, *RXRA, TFR2*, *TTC21B* *
[18]	1 (1 adult patient with cholestasis, part of a family trio)	WES performed on family trio (father, mother, child)	Proband with 2 *ATP8B1* variants	-	*ATP8B1*	**-**
[19]	1	WES	Proband with 1 *ABCB4* variant	**-**	*ABCB4*	**-**
[20]	52	WES	23% (12/52)	11.5% (6/52)	*ABCB4*, *ABCB11*, *APOB*, *JAG1*, *RTEL1*, *SCARB1*, *SLC40A1*, *SMAD3*, *HNF1B*	*ABCB4*, *ABHD5*, *DYSF*, *LIPE*
[21]	1	WGS	Proband with 2 *USP53* variants	**-**	*USP53*	**-**
[22]	1	WES	Proband with 2 *ABCB4* variants	-	*ABCB4*	-
[23]	1	Targeted NGS panel (61 genes responsible for genetic disorders of hepatic cholestasis)	Proband with 1 *ATP8B1* variant	-	*ATP8B1*	-
[24]	1	Targeted NGS panel (61 genes responsible for genetic disorders of hepatic cholestasis)	Proband with 2 *ABCB4* variants	-	*ABCB4*	-
[25]	4	WES to detect *ABCB4* variants	100% (4/4)	-	*ABCB4*	
[5]	356	Targeted NGS panel (3 genes)	7% (25/356)	12% (43/356)	*ATP8B1*, *ABCB11*, *ABCB4*	*ATP8B1*, *ABCB11*, *ABCB4*
[26]	1	Targeted NGS panel (76 genes within the Genetic Cholestasis panel)		Proband with 2 *ABCB4* variants		*ABCB4*
[27]	1	Targeted NGS panel (9 genes known to be involved in a spectrum of liver and cystic diseases)	1 homozygous *TJP2* variant	-	*TJP2*	-
[4]	45	Targeted NGS panel (4 genes)	22% (10/45)	15.5% (7/45)	*ATP8B1*, *ABCB11*, *ABCB4*, *TJP2*	*ATP8B1*, *ABCB11*, *ABCB4*, *TJP2*
[28]	2	Targeted NGS panel (3 genes)	100% (2/2)	-	*ABCB11*	-

**Table 4:** **Gene symbols associated with adult-onset cholestatic liver diseases, their full names, and functional relevance detected by next-generation sequencing and whole-exome sequencing: challenges in interpreting variants of uncertain significance.**

Gene Symbol	Full Name	Function and Relevance
***ABCB11***	ATP Binding Cassette Subfamily B Member 11	Encodes BSEP, a bile salt export pump; mutations cause PFIC2 and are associated with cholestatic liver disease.
***ABCB4***	ATP Binding Cassette Subfamily B Member 4	Encodes MDR3, a phospholipid transporter in the liver; mutations cause PFIC3 and are linked to LPAC and ICP.
***ABCC2***	ATP Binding Cassette Subfamily C Member 2	Encodes MRP2, a transporter involved in bile excretion; mutations cause Dubin–Johnson syndrome.
***ABHD5***	Abhydrolase Domain-Containing Protein 5	It encodes an activator of lipolysis, which has been linked to autosomal recessive Chanarin–Dorfman syndrome, a neutral lipid storage disease characterized by myopathy, ichthyosis, and fatty liver.
***AGL***	Amylo-1,6-Glucosidase, 4-Alpha-Glucanotransferase	Involved in glycogen metabolism; mutations cause glycogen storage disease type III.
***APOB***	Apolipoprotein B	Key component of lipoproteins; mutations can affect lipid metabolism and liver function.
***ATP8B1***	ATPase Phospholipid Transporting 8B1	Encodes FIC1, involved in bile acid transport; mutations cause PFIC1 and related cholestatic disorders.
***CP***	Ceruloplasmin	Copper-binding protein; mutations cause Wilson disease and can affect liver health.
***DCDC2***	Doublecortin Domain Containing 2	Associated with ciliary function; mutations linked to neonatal sclerosing cholangitis and hepato-renal ciliopathies.
***DYSF***	Dysferlin	Encodes Dysferlin, highly expressed in muscle tissue and essential for muscle membrane repair.
***FOCAD***	Focadhesin	Encodes Focadhesin, a poorly characterized protein important for cell adhesion and motility. It is also involved in ciliary function and, if altered, it can cause severe liver disease and developmental defects in the bile ducts.
***HFE***	High Fe (Iron)	Encodes a protein involved in regulating intestinal iron absorption. It acts as a sensor of circulating iron and stimulates the production of hepcidin, a liver hormone that inhibits iron release from the intestine and macrophages, maintaining iron homeostasis.
***HNF1B***	HNF1 Homeobox B	Regulates the expression of numerous genes during embryonic development and the function of various organs, particularly important in the liver.
***JAG1***	Jagged 1	Binds to Notch receptors (especially NOTCH2) on nearby cells, activating the Notch pathway that is essential during embryonic development of liver and bile ducts. Pathogenic variants of JAG1 cause Alagille syndrome.
***KLF11***	KLF Transcription Factor 11	Associated with MODY-7.
***LIPE***	Lipase, Hormone-Sensitive	Encodes lipase E, a hormone-sensitive lipase that plays a role in the rate-limiting step of fatty acid breakdown and mobilization from triglycerides.
***MBOAT7***	Membrane-Bound O-Acetyltransferase Domain-Containing Protein 7	Encodes an enzyme involved in membrane lipid metabolism, remodeling membrane phospholipids; if altered by steatosis, fibrosis, or progression to cirrhosis, even in patients without classical causes.
***MTTP***	Microsomal Triglyceride Transfer Protein	Involved in lipid transport; mutations can cause abetalipoproteinemia and liver disease.
***NPHP3***	Nephrocystin 3	Associated with nephronophthisis and ciliopathies; can contribute to hepato-renal syndromes.
***PKHD1***	Polycystic Kidney and Hepatic Disease 1	Mutations cause autosomal recessive polycystic kidney disease, often with liver involvement.
***PPOX***	Protoporphyrinogen Oxidase	Involved in heme biosynthesis; mutations cause variegate porphyria, which can affect the liver.
***TJP2***	Tight Junction Protein 2	Encodes a protein important for tight junctions in hepatocytes; mutations cause PFIC4 and are linked to cryptogenic cholestasis and cirrhosis.
***RTEL1***	Regulator of Telomere Elongation Helicase 1	Encodes a DNA helicase that stabilizes and lengthens telomeres during DNA replication. If alterated, it can cause lung and liver fibrosis.
***SCARB1***	Scarvenger Receptor Class B, member 1	Implicated in HDL cholesterol regulation and foam cell stabilization in atherosclerosis.
***SLC40A1***	Solute Carrier Family 40, member 1	Encodes Ferroportin and, if alterated, it can cause Hereditary Hemochromatosis Type 4.
***SMAD3***	SMAD Family member 3	Related to Loeys–Dietz syndrome type 3, associated with arterial aneurysms, vascular tortuosity, and valvular insufficiency.
***TFR2***	Transferrin Receptor 2	Encodes Transferrin Receptor 2, a protein critical for controlling iron homeostasis. Biallelic pathogenic variants of TFR2 cause Hereditary Hemochromatosis Type 3.
***TMEM199***	Transmembrane Protein 199	Related to Congenital Disorder of Glycosylation, Type IIp.
***TTC21B***	Tetratricopeptide Repeat Domain 21B	Related to ciliary function; mutations implicated in cholestatic liver disease.
***USP53***	Ubiquitin Specific Peptidase 53	Involved in tight junction integrity; mutations cause PFIC7, often with low-GGT cholestasis.
***VPS33B***	Vacuolar Protein Sorting 33B	Involved in vesicle trafficking; mutations cause ARC syndrome, which includes liver dysfunction.

## Data Availability

No new data were created or analyzed in this study. Data sharing is not applicable to this article.

## Supplementary Materials

The following supporting information can be downloaded at: https://www.mdpi.com/article/10.3390/diagnostics16152335/s1.

## Abbreviations

The following abbreviations are used in this manuscript:

ACMG	American College of Medical Genetics and Genomics
BRIC	Benign Recurrent Intrahepatic Cholestasis
GGT	Gamma-Glutamyl Transferase
IBAT	Ileal Bile Acid Transporter Inhibitors
ICP	Intrahepatic Cholestasis of Pregnancy
LP	Likely Pathogenic variants
LPAC	Low Phospholipid-Associated Cholelithiasis
NGS	Next-Generation Sequencing
NR	Not reported
OMIM	Online Mendelian Inheritance in Man
P	Pathogenic variants
PFIC	Progressive Familial Intrahepatic Cholestasis
PSC	Primary Sclerosing Cholangitis
UDCA	Ursodeoxycholic acid
VUS	Variants of Uncertain Significance
WES	Whole-Exome Sequencing
WGS	Whole-Genome Sequencing
