# Supplementary material for: Use of Next-Generation Sequencing and Whole-Exome Sequencing in the Diagnosis of Adult-Onset Familial Intrahepatic Cholestasis: Challenges in Interpreting Variants of Uncertain Significance
Source: Diagnostics (Basel). 2026 Jul 25;16(15):2335. doi: 10.3390/diagnostics16152335 (PMC13464336; doi:10.3390/diagnostics16152335)
Supplement: Supplementary file 1 [file diagnostics-16-02335-s001.zip › diagnostics-4205642-supplementary.pdf]

Reference	Pz n.	Gene	Variant Description	Zigosity	Variant Type	Classification ACMG	Phenotypic Association
[11]	1	*ABCB4*	c.2177C>T p.Pro726Leu	Het	Missense	Pathogenic	Pregnancy-related cholestasis, cholecystitis, biliary pancreatitis, chronically elevated cholestatic enzymes
[11]	2	*ABCB4*	c.716C>T p.Ser239Leu	Hom	Missense	Likely Pathogenic	Age 18, elevated ALT/AST/ALP/GGT, normal US, mixed portal inflammation; normalized after UDCA
[11]	3	*AGL*	c.889A>T p.Lys297Ter	Hom	Missense	Pathogenic	Cirrhosis, proximal muscle weakness, elevated CK, vacuolar myopathy, glycogen storage disease type IIIa
[11]	4	*APOB*	c.29C>T p.Ala10Val	Hom	Missense	VUS	Hypercholesterolemia, pepati steatosis
[11]	4	*APOB*	c.2258G>A p.Gly753Glu	Hom	Missense	VUS	Hypercholesterolemia, pepati steatosis
[11]	4	*CP*	c.1948G>A p.Gly650Arg	Het	Missense	Pathogenic	Low ceruloplasmin, steatohepatitis F1–F2, essential tremors, dystonia, diabetes
[11]	4	*KLF11*	c.42 + 3G > C	Het	Intronic variant	VUS	Same patient as above
[11]	5	*MTTP*	c.59_61+14del	Compound Het	Deletion	Likely Pathogenic	Cirrhosis, low ApoB/LDL/TG, chronic diarrhea, low vitamins, osteoporosis, night blindness
[11]	5	*MTTP*	c.1946A>G p.Asn649Ser	Compound Het	Missense	Likely Pathogenic	Same patient as above
[11]	6	*VPS33B*	c.277C > T p.Arg93*	Het	Nonsenso	Pathogenic	Idiopathic cholestasis, family history positive
[12]	1	*ABCB4*	c.754G>C p.Ala252Pro	Compound Het	Missense	Likely Pathogenic	Recurrent lithiasis
[12]	1	*ABCB4*	c.2144C>T p.Thr715Ile	Compound Het	Missense	VUS	Recurrent lithiasis
[12]	2	*ABCB4*	c.526C>T p.Arg176Trp	Het	Missense	Pathogenic	Recurrent lithiasis
[12]	3	*ABCC2*	c.468+1G>A	Hom	Splicing	Likely Pathogenic	Intrahepatic cholestasis
[12]	4	PPOX	c.338+2dup	Het	Duplication	Likely Pathogenic	Sclerosing cholangitis, unusual features
[12]	5	APOB	c.13480_13482del p.Gln4494del	Het	Deletion	Likely Pathogenic	Sclerosing cholangitis + steatotic liver disease
[13]	1	*ATP8B1*	c.1710_1711insCCTC p.Ala571Profs*12	Compound Het	Frameshift	Pathogenic	Chronic cholestasis, low GGT, jaundice, itching, impaired bile flow, family history
[13]	1	*ATP8B1*	c.2989G>A p.Ala997Thr	Compound Het	Missense	Likely Pathogenic	Same patient as above
[14]	1	*ABCB4*	c.2784-12T>C	Hom	Intronic	Pathogenic	Hepatomegaly, jaundice, chronic cholestasis, impaired bile flow
[14]	2	*ABCB4*	c.1633C>T p.Arg545Trp	Het	Missense	Pathogenic	Chronic cholestasis, persistent jaundice/pruritus, alcohol history

[15]	1	*USP53*	c.972+3_972+6del	Compound Het	Deletion	Likely Pathogenic	Chronic cholestatic hepatitis, low GGT, mild fibrosis, hepatomegaly, intermittent jaundice/pruritus
[15]	1	*USP53*	c.973–1G>A	Compound Het	Splicing	Likely Pathogenic	Same patient as above
[16]	1	*ABCB4*	c.1445C>T p.Arg482Trp	Het	Missense	Pathogenic	Adult cholestasis, pruritus/jaundice, cirrhosis, low/normal GGT, elevated ALT/AST/bilirubin
[17]	1	*ATP8B1*	c.1429+2T>G	Het	Splicing	Pathogenic	Recurrent jaundice/pruritus, BRIC1, normal GGT, no progression to chronic liver disease
[18]	1	*ABCB4*	c.959C > T p.Ser320Phe	Compound Het	Missense	Pathogenic	Severe pruritus/jaundice, end-stage liver disease, liver transplant; post-transplant improvement
[18]	1	*ABCB4*	c.1429C>A p.Thr477Asn	Compound Het	Missense	Pathogenic	Same patient as above
[19]	1	*ABCB4*	c.355G>A p.Ala119Thr	Compound Het	Missense	Pathogenic	Chronic ductopenia in liver graft 15 years post-transplant
[19]	1	*ABCB4*	c.1445C>T p.Thr482Met	Compound Het	Missense	Pathogenic	Same patient as above
[20]	1	*TJP2*	c.3334C >T p.Gln1112*	Hom	Nonsense	Pathogenic	Advanced cirrosi, variceal bleeding. HCC, liver transplant at 20, consanguineous family, variable severity in sibling
[4]	1	*ATP8B1*	c.3655G>C p.Asp1219His	Het	Missense	Likely Pathogenic	Adult woman, mild biochemical abnormalities, FibroScan 5.5 kPa
[4]	1	*TJP2*	c.1057C>T p.Arg322Trp	Het	Missense	Likely Pathogenic	Same patient as above
[4]	2	*ATP8B1*	c.68C>T p.Pro23Leu	Het	Missense	Likely Pathogenic	Juvenile cholelithiasis, neonatal jaundice, positive family history
[4]	3	*ABCB11*	c.278A>C p.Tyr93Ser	Compound Het	Missense	Likely Pathogenic	Neonatal jaundice, DIC, ICP, juvenile cholelithiasis, itching, normal GGT
[4]	3	*ABCB11*	c.1789G>C p.Val597Leu	Compound Het	Missense	Likely Pathogenic	Same patient as above
[4]	3	*ABCB11*	c.3382C>T p.Arg1128Cys	Compound Het	Missense	Pathogenic	Same patient as above
[4]	4	*ABCB11*	c.1568C>G p.Ala523Gly	Het	Missense	Likely Pathogenic	Adult-onset cholestasis, pruritus, neonatal jaundice, high FibroScan
[4]	5	*ABCB11*	c.403G>A p.Glu135Lys	Compound Het	Missense	Likely Pathogenic	Itching, neonatal jaundice, listed for liver transplant

[4]	5	*ABCB11*	c.3297del p.Ser1100Glnfs*38	Compound Het	Frameshift	Pathogenic	Same patient as above
[4]	6	*ABCB4*	c.2014A>T p.Lys672*	Het	Nonsense	Likely Pathogenic	39-year-old woman, family history of cholestatic disease (father affected, high GGT), history of DIC and itching
[4]	7	*ABCB4*	c.1091C>T p.Ala364Val	Het	Missense	Likely Pathogenic	57-year-old cirrhotic patient with DIC and hepatocellular carcinoma
[4]	8	*TJP2*	c.185C>T p.Thr62Met	Het	Missense	Likely Pathogenic	51-year-old woman, GGT 84 U/L, ALP 205 U/L, bilirubin 0.6 mg/dL, FibroScan 4.4 kPa; DIC and ICP; no affected relatives; segregation not assessed
[4]	9	*TJP2*	c.185C>T p.Thr62Met	Het	Missense	Likely Pathogenic	50-year-old man, GGT 50 U/L, ALP 130 U/L, AST 175 U/L, bilirubin 0.6 mg/dL, FibroScan 3.3 kPa; no affected relatives; segregation not assessed
[4]	10	*TJP2*	c.2717T>C p.Ile875Thr	Het	Missense	Likely Pathogenic	37-year-old man, GGT 217 U/L, ALP 399 U/L, bilirubin 0.6 mg/dL, FibroScan 8.1 kPa; positive family history; segregates in affected brother with elevated GGT
[21]	1	*ABCB11*	c.403G>A p.Glu135Lys	Compound Het	Missense	Likely Pathogenic	Adult-onset cholestasis with pruritus, mild jaundice, elevated liver enzymes; intermediate phenotype between BRIC2 and PFIC2
[21]	1	*ABCB11*	c.3298_3299del p.Ser1100Glnfs*38	Compound Het	Frameshift	Likely Pathogenic	Same patient as above
[21]	2	*ABCB11*	c.1567G>A p.Ala523Gly	Hom	Missense	Likely Pathogenic	Adult-onset cholestasis with pruritus and moderate biochemical abnormalities; milder intermediate phenotype compared to classical pediatric PFIC2

Supplementary Table S1. List of pathogenic and likely pathogenic variants identified by NGS panel, WES or WGS and related hepatic disorders in adult patients affected by cryptogenic Cholestasis in the articles published up to today (Het= heterozigous; Hom= homozigous)
